# Meetings of Societies

**Published:** 1910-12

**Authors:** 


### MEETINGS OF SOCIETIES.

### Bristol Medico-Chirurgical Society.

*Annual Meeting, October 12th,* 1910.

After a vote of thanks to the retiring President, Dr. James Swain, had been passed, Dr. B. M. H. Rogers took the chair and gave an introductory address (see p. 289). Later a cordial vote of thanks was given to Dr. Rogers for his able and interesting address.

The Honorary Secretary and Editorial Secretary, Dr. J. A. Nixon, read the following annual report :—

There is a balance in hand on the General Account of £126. From members' subscriptions the receipts were £164 17s. 0d. Balance from previous year £81 14s. 8d. Interest on deposit account £2 0s. 9d. The expenses amounted to £122 16s. 11d. It should be noted that the balance in hand is artificially inflated by the fact that the Library Grant has not yet been paid.

The balance in hand on the *Journal* Account at the end of 1909 was £2 5s. 2d.

During the year 1909–10 eighteen members joined the Society and eight resigned. We have to deplore the death of two ex-Presidents, Mr. Coe and Dr. Spencer. On October 1st, 1910, there were 171 members.

The Honorary Librarian, Mr. C. K. Rudge, read the following report :—

The Library of the Bristol Medico-Chirurgical Society now contains 11,390 volumes. During the year 292 volumes have been added. The Library is in regular receipt of 190 periodicals.

During the year 1,107 issues of books have been loaned to 105 borrowers. Our membership of Lewis's Library has proved of such service that our subscription has been doubled, and we now have the use of 14 volumes at a time. During the past twelve months 65 volumes were obtained from that Library.

The Bristol Medical Library, to which members of the Society have access, now consists of 21,630 volumes, and is in receipt of 249 periodicals. These are contributed by the constituent libraries in the following manner :—

BOOKS.

| | |
|---|---|
| Bristol Medico-Chirurgical Society .. .. | 11,390 |
| Faculty of Medicine, University .. .. | 8,477 |
| Infirmary .. .. .. .. .. .. .. .. | 1,189 |
| General Hospital .. .. .. .. .. .. | 574 |
| Total .. .. .. .. | 21,630 |

PERIODICALS.

| | |
|---|---|
| Medico-Chirurgical Society .. .. .. | 190 |
| Faculty of Medicine .. .. .. .. .. .. | 59 |
| Total .. .. .. | 249 |

The following are the officers of the Society for the Session 1910–11: President—Bertram M. H. Rogers, B.A., M.D. Oxon. President-elect—C. A. Morton, F.R.C.S. Committee (*Ex-Officio*)—James Swain, M.D., M.S. Lond. (retiring President); R. Shingleton Smith, M.D. Lond. (Editor of *Journal*); C. K. Rudge, M.R.C.S. (Hon. Librarian); J. A. Nixon, B.A., M.B. Cantab. (Editorial Secretary). (*Elected*)—J. Taylor, M.R.C.S. (Ex-President); J. Michell Clarke, M.A., M.D. Cantab. (Ex-President); P. Watson Williams, M.D. Lond.; G. Parker, M.D. Cantab.; I. Walker Hall, M.D. Vict.; F. Lace, F.R.C.S.; J. Lacy Firth, M.D., M.S. Lond.; John Wallace, M.B., C.M., B.Sc. Edin. Hon. Secretary—J. A. Nixon, B.A., M.B. Cantab.

**RECEIPT AND EXPENDITURE ACCOUNT OF THE BRISTOL MEDICO-CHIRURGICAL SOCIETY, SESSION 1909-10.**

| | £ | s. | d. | | £ | s. | d. |
|---|---|---|---|---|---|---|---|
| 1909. | | | | 1909. | | | |
| Oct. 1.—Balance in hand | 81 | 14 | 8 | Oct. 14.—Bank Postages | 0 | 0 | 9 |
| ,, 1.—Members' Subscriptions .. | 164 | 17 | 0 | Nov. 5.—Honorarium and Stamps | 6 | 5 | 0 |
| 1910. | | | | 1910. | | | |
| Sept. 20.—Interest on Deposit Account | 0 | 9 | 2 | Jan. 11.—Bromhead .. | 1 | 1 | 0 |
| ,, 20.—Interest on Deposit Account | 1 | 11 | 7 | ,, 11.—Stamps.. .. | 1 | 0 | 0 |
| ,, 22.—Cheque not presented .. | 0 | 5 | 0 | ,, 11.—Lantern .. | 1 | 1 | 0 |
| Oct. 1.—Petty Cash .. | 0 | 18 | 9 | Apr. 1.—Stamps.. .. | 1 | 0 | 0 |
| | | | | ,, 19.—Lantern .. | 1 | 1 | 0 |
| | | | | July 6.—Editorial Sec. | 47 | 15 | 0 |
| | | | | Aug. 16.—Porter .. .. | 1 | 5 | 11 |
| | | | | Sept. 10.—Rent, etc. .. | 57 | 6 | 0 |
| | | | | ,, 20.—Printing .. | 2 | 14 | 6 |
| | | | | ,, 22.—Typewriting | 0 | 5 | 0 |
| | | | | ,, 27.—Repairs .. | 1 | 3 | 0 |
| | | | | Hon. Sec.'s Petty Cash .. .. | 0 | 18 | 9 |
| | | | | Balance in Bank | 126 | 19 | 3 |
| | £249 | 16 | 2 | | £249 | 16 | 2 |

---

*November* 9th, 1910.

Dr. Bertram M. H. Rogers, President, in the Chair.

Dr. J. O. Symes read notes of a case of **Myasthenia with Enlargement of the Thymus.** The patient was a woman of 21 who had been taken ill, ten days before admission to hospital, with pain in the head and neck, followed in two or three days by weakness of the legs. Also difficulty in swallowing was then noticed. Later there had been tingling in the hands with pain and loss of power in both arms, a rapid pulse, and occasional attacks of heart failure. On admission to hospital the patient was much exhausted. She was hysterical in manner, there was marked weakness of the limbs, and the patient could not stand. Ptosis of both eyelids was present. Mastication and swallowing were difficult. No wasting. Thirty hours after admission the patient became restless and dyspnœic, and died suddenly three-quarters of an hour later. A *post-mortem* had been made by Dr. Emrys-Roberts. Both lobes of the thymus were much enlarged, also the thyroid was somewhat enlarged. The duodenum showed a number of dilated capillaries, and the ileum showed the same and also several hemorrhagic areas. The liver and pancreas showed similar hemorrhages. The speaker considered the diagnosis, and gave reasons for considering the case to be one of **myasthenia gravis.** A difficulty was the rapidity of the progress of the disease. Campbell and Bramwell had found the average duration of myasthenia in sixty cases to be one and a half years, but one case had ended fatally in fourteen days and another in a month. The pathological changes found at the *post-mortem* supported the diagnosis. Buzzard's statement was quoted that the symptoms of the disease "are best explained by assuming the presence of some toxic agent which has a special influence on the protoplasmic (as opposed to the fibrillar) constituent of voluntary muscle."

Dr. W. H. C. Newnham read a paper on **225 Consecutive Laparotomies with Nine Deaths.** The cases were grouped into twelve classes, and the causes of death in the fatal cases considered. Also some accidents and complications which have been met with were described, including one case in which an end to end suture of torn bowel had been successfully accomplished.

Dr. E. C. Williams read a paper on **The Treatment of six cases of Posterior Basic Meningitis.** During the past two and a half years six cases of the disease had been under his care, three cases were under twelve months, and three were between one and two years of age. Three children were males, three females. All the cases occurred during the first nine months of the year. Lumbar puncture was performed in each. In two cases, both of which recovered, lumbar puncture alone was performed, one child, however, was an idiot. Of the four cases treated with lumbar puncture and Ruppil's serum, three recovered, one died. Lumbar puncture is useful both from a diagnostic and therapeutic standpoint. In three cases the fluid was cloudy, and in two cases diplococci were present; in one case undoubtedly the diplococcus cellularis was present, in the other a diplococcus which was gram-positive. A gram-positive diplococcus has been found in other cases of this disease. Lumbar puncture is only one element in the diagnosis, and its findings must be taken in conjunction with established clinical signs. The child's weight is the surest and earliest guide as to the efficacy of treatment. Dose of serum for an injection varies from $1\frac{1}{2}$ to 5 cc. As to the number of injections one must be guided by the progress of the case. Whether it is possible to differentiate between the diplococcus found in this disease and the diplococcus of cerebro-spinal meningitis is a question which has not yet been finally decided. Serum treatment certainly seems to afford the best chances for a complete recovery in this disease. Dr. EDGEWORTH remarked upon the value of the suggestion, an original one he believed, that the increase or decrease in weight gave a very important clue to prognosis in cases of this disease.

Mr. C. A. MORTON showed a specimen of **Twin Gestation in one Fallopian Tube.**

J. LACY FRITH.
J. A. NIXON, *Hon. Sec.*

